# Four-Dimensional Sequential Elastography for Ocular Tissue Using 2D Matrix Array

**DOI:** 10.34133/bmef.0307

**Published:** 2026-09-09

**Authors:** Xin Sun, Yushun Zeng, Matthew Xinhu Ren, Xiaoyang Yu, Chi-Feng Chang, Xunan Liu, Junhang Zhang, Ying Liu, Bo Wang, Yaoheng Yang, Brian J. Song, Martin Heur, Mark S. Humayun, Qifa Zhou

**Affiliations:** ^1^ Alfred E. Mann Department of Biomedical Engineering, University of Southern California, Los Angeles, CA, USA.; ^2^ USC Roski Eye Institute, Department of Ophthalmology, Keck School of Medicine, University of Southern California, Los Angeles, CA, USA.; ^3^ Ming Hsieh Department of Electrical and Computer Engineering, University of Southern California, Los Angeles, CA, USA.

## Abstract

**Objective:** This study aims to facilitate a sequential excitation 4-dimensional (4D) ultrasound shear wave elastography (SWE) method for volumetric characterization of mechanical properties of ocular tissue using a 2D matrix array with a standard 256-channel ultrasound system. **Impact Statement:** Sequential elastography (SE) enables cost-effective 4D volumetric elastography without requiring an equal channel-count system. The swept-cross strategy improves volumetric imaging quality and, for the first time, enables 4D SWE-based differentiation of tissue anisotropy, with in vivo ocular applications. **Introduction:** Volumetric SWE remains challenging because of the limited channel counts of the acquisition system. **Methods:** SE applies a 32 × 32 matrix array and divides it into four 8 × 32 subpanels, connected with a Vantage 256 system through 4-1 multiplexing. An optimized sequential excitation and swept-cross compounding method is used to reduce the interpanel gap influence and improve elevational performance. **Results:** Validated using wire targets, point target, ex vivo chicken breast, and in vivo rabbit cornea, SE improved volumetric imaging performance compared with conventional subpanel compounding. It improved the elevational resolution from 0.84 mm to 0.35 mm at 10-mm depth and the 3D point spread function from 1 × 2.1 × 4.7 *λ*^3^ to 1 × 2.2 × 2.1 *λ*^3^. In chicken breast, it differentiated tissue anisotropy and estimated muscle fiber direction. In rabbit cornea, it enabled volumetric shear wave velocity mapping. **Conclusion:** SE enables 4D shear wave elastography and provides a promising approach for volumetric assessment of tissue biomechanics, anisotropy, and ocular stiffness.

## Introduction

Shear wave elastography (SWE) has seen considerable development and is now commonly used in clinics to provide non-invasive, quantitative mechanical information of biological tissue [1–3]. By providing the mapping of tissue stiffness, SWE has emerged as a useful complementary imaging technique for tissue characterization and disease assessment [4–8], especially in liver failure [9], breast lesion differentiation [10], and diagnosis of thyroid nodules [11]. However, current clinical implementations of SWE are restricted by 2-dimensional (2D) imaging, largely due to the limited channel count of ultrasound acquisition systems and the high computational cost required for volumetric imaging [12–14]. Furthermore, because biological tissues have complex, directional structures (anisotropy), conventional 2D SWE cannot fully capture the mechanical properties within the region of interest. This limitation becomes particularly important in biological tissues with anisotropic structures, where mechanical properties may vary with spatial direction [14,15].

To address these limitations of 2D SWE, recent advances in ultrasound elastography have rapidly shifted toward volumetric imaging, specifically 3D SWE [16–18]. Volumetric elastography provides a comprehensive spatial map of tissue stiffness, enabling more precise localization and characterization of pathological structures [19,20]. The earliest 3D SWE approaches relied on mechanically swept or wobbler probes [21–23]. However, such mechanical methods are limited by extremely poor temporal resolution and cannot capture the true transient propagation of shear waves in 3D space [24,25].

Consequently, the coupling of 2D matrix arrays with ultrafast volume imaging is widely recognized for effectively achieving true 4D (*x*, *y*, *z*, *t*) SWE [26,27]. Driving a 2D matrix array (e.g., a 1,024-element array) typically requires 4 synchronized 256-channel ultrasound systems operating in parallel to achieve ultrafast volume rates [16,26]. Although 2D matrix array transducers have demonstrated great potential for 4D SWE, their application is still limited by hardware complexity and cost [25]. Therefore, recent studies have focused on adapting volumetric elastography to a single 256-channel ultrasound system. For example, row-column addressing (RCA) arrays have been proposed to reduce the channel-count requirement [28,29]. Both acoustic radiation force (ARF)-based and mechanically excited SWE have demonstrated promising results with RCA arrays [29]. However, due to the limitation in beamforming strategy [25], 3D spatial resolution remains limited, which restricts RCA-based volumetric SWE to differentiate tissue anisotropy.

To enable 4D SWE using a 1,024-element 2D matrix array on a 256-channel ultrasound acquisition system, we previously proposed a novel 4D ultrasound SWE method based on sequential excitation (SE-SWE) [30]. SE-SWE employs multiplexing to divide the 1,024-element 2D matrix array into 4 independent sub-apertures. Using a standard 256-channel ultrasound system, each sub-aperture is sequentially excited to capture shear wave propagation [31]. The resulting subvolumes are subsequently reconstructed and combined to form the complete volumetric shear wave field. Through this approach, we successfully demonstrated high-quality 4D shear wave tracking and velocity reconstruction while substantially reducing hardware costs.

However, manufacturing complexities mean that commercially available 1,024-element 2D matrix arrays (such as the Vermon 1,024-element array) are typically assembled from multiple independent piezoelectric panels rather than a single monolithic structure, which can introduce uncompensated spatial offsets and phase distortions [32].

In this study, we optimized a sequential excitation scheme to mitigate wavefront aberrations and the influence of geometric discontinuities caused by the large gap between array panels. ARF-based SWE experiments on chicken breast tissue demonstrated that the proposed method can differentiate tissue anisotropy and accurately estimate muscle fiber orientation. Furthermore, in vivo rabbit experiments further validated its capability for volumetric elastography mapping and demonstrated the first successful shear wave velocity mapping of ocular tissue.

## Results

### Sequential excitation for 3D shear wave detection

**Fig. 1.**
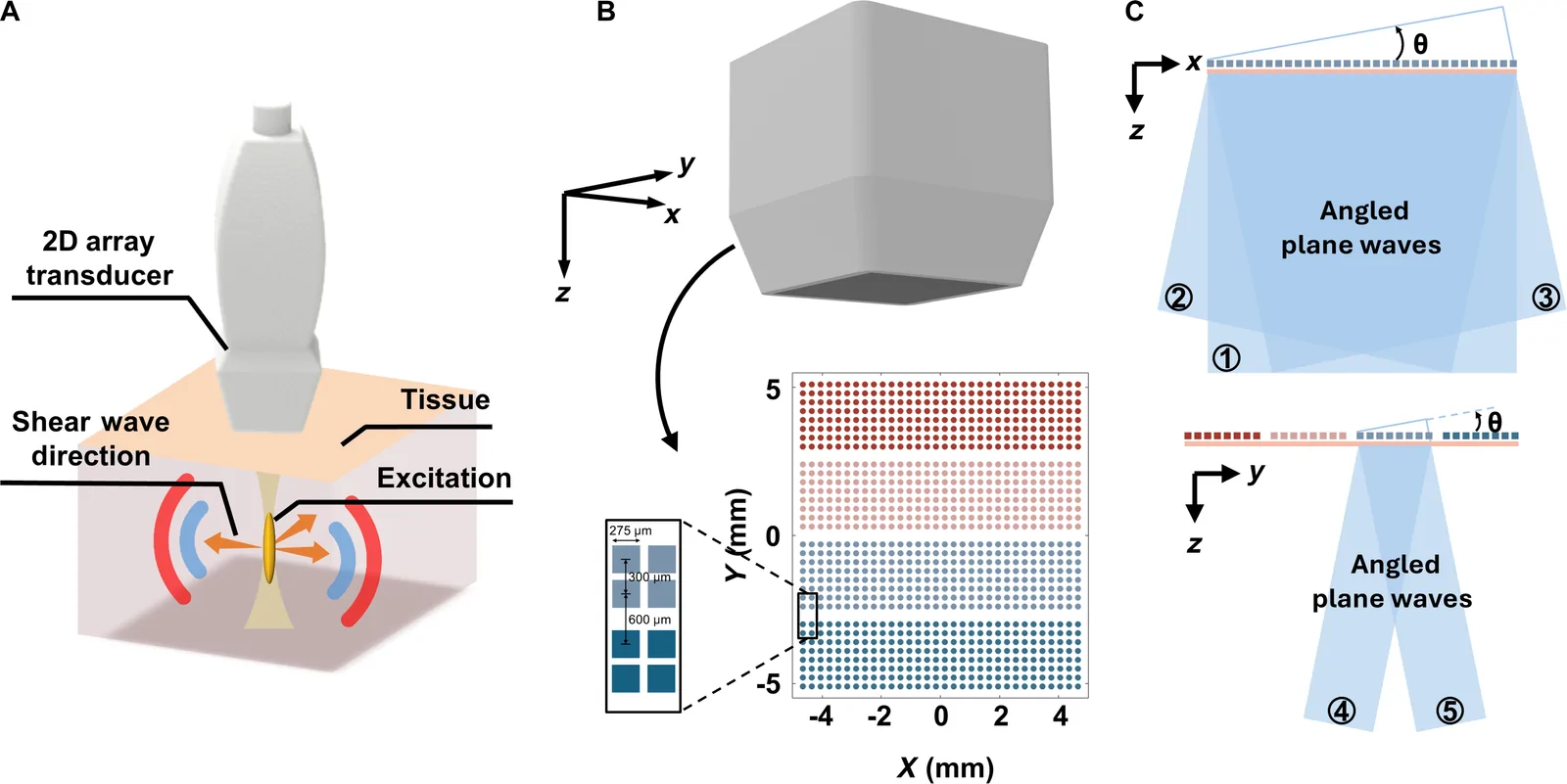
Schematic diagram of 3D SWE and transmit–receive strategy. (A) Schematic illustration of 3D shear wave propagation. (B) Schematic diagram of the 2D matrix array structure, divided into four 8 × 32 subpanels. (C) Schematic illustration of coherent compounding plane wave transmission and receive.

The principle of 3D shear wave detection is illustrated in Fig. 1A. The induced shear waves propagate through the tissue as 3D waves when exciting ARF or mechanical excitation. To accurately capture the complex spatial-temporal dynamics of shear waves, a 1,024-element 2D matrix array with a center frequency of 8 MHz was applied. The array was divided into 4 subpanels (8 × 32 elements each) and connected to a 256-channel programmable ultrasound system via a 4-1 multiplexing, as shown in Fig. 1B.

### Wire phantom and point target results

**Fig. 2.**
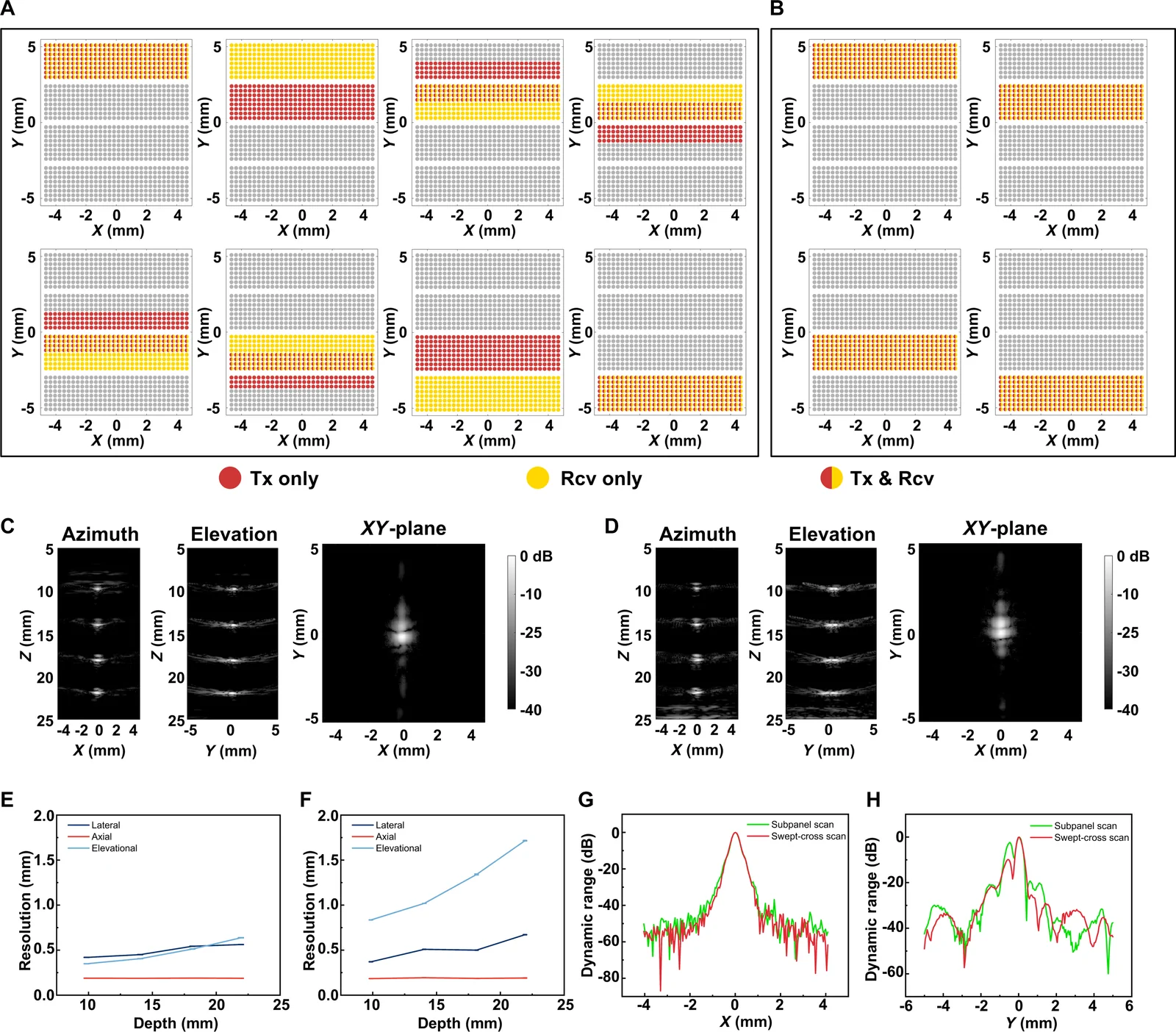
Schematic illustration of the fast cross-panel acquisition sequence and corresponding resolution results. (A and B) Schematic comparison between the fast swept-cross scan sequence and conventional subpanel compounding. (C and D) B-mode imaging results of wire and point targets. (E to H) 3D resolution results. Fast swept-cross scan: 1 × 2.2 × 2.1 *λ*^3^. Conventional subpanel compounding: 1 × 2.1 × 4.7 *λ*^3^ at depth 10 mm.

Volumetric plane-wave imaging was performed using 5 steering angles (0°, 0°), (−5°, 0°), (5°, 0°), (0°, −5°), and (0°, 5°). Element positions were calibrated prior to the experiment [32]. To mitigate the detrimental effect of the large gap between the subpanels, as shown in Fig. 1B, a swept-cross compounding strategy was implemented. Specifically, 8 transmission–receive groups were designed to improve image quality, as illustrated in Fig. 2A. This method was compared with the previously proposed sequential excitation compounding approach based on conventional 4-panel transmission–receive acquisition, as shown in Fig. 2B [30]. The B-mode imaging results for the wire target and point target are presented in Fig. 2C and D, showing the expected imaging quality of the 1,024-element 2D array. Figure 2E and F compared the spatial resolution under 2 different transmission–receive sequences. Based on the imaging results of the wire target and point target, the swept-cross sequence significantly improved the elevational resolution by calculating the full width at half maximum (FWHM). In the near field, at a depth of 10 mm, the lateral resolution improved from 0.84 mm to 0.35 mm, corresponding to a 58.3% reduction. The contrast-to-noise ratio (CNR) increased from 4.5802 to 7.4371. At depths of 14 and 18 mm, the elevational resolution improved from 1.0 mm to 0.4 mm and from 1.3 mm to 0.5 mm, corresponding to improvements of 60.0% and 61.5%, respectively. At a depth of 22 mm, resolution improved from 1.7 mm to 0.63 mm, a 62.9% reduction. The swept-cross sequence yielded a 3D point spread function (PSF) of 1×2.2×2.1 *λ*^3^, compared with 1×2.1×4.7 *λ*^3^ obtained using 4-panel transmission–receive acquisition, as shown in Fig. 2G and H.

### Predicting muscle fiber orientation via 4D SWE

**Fig. 3.**
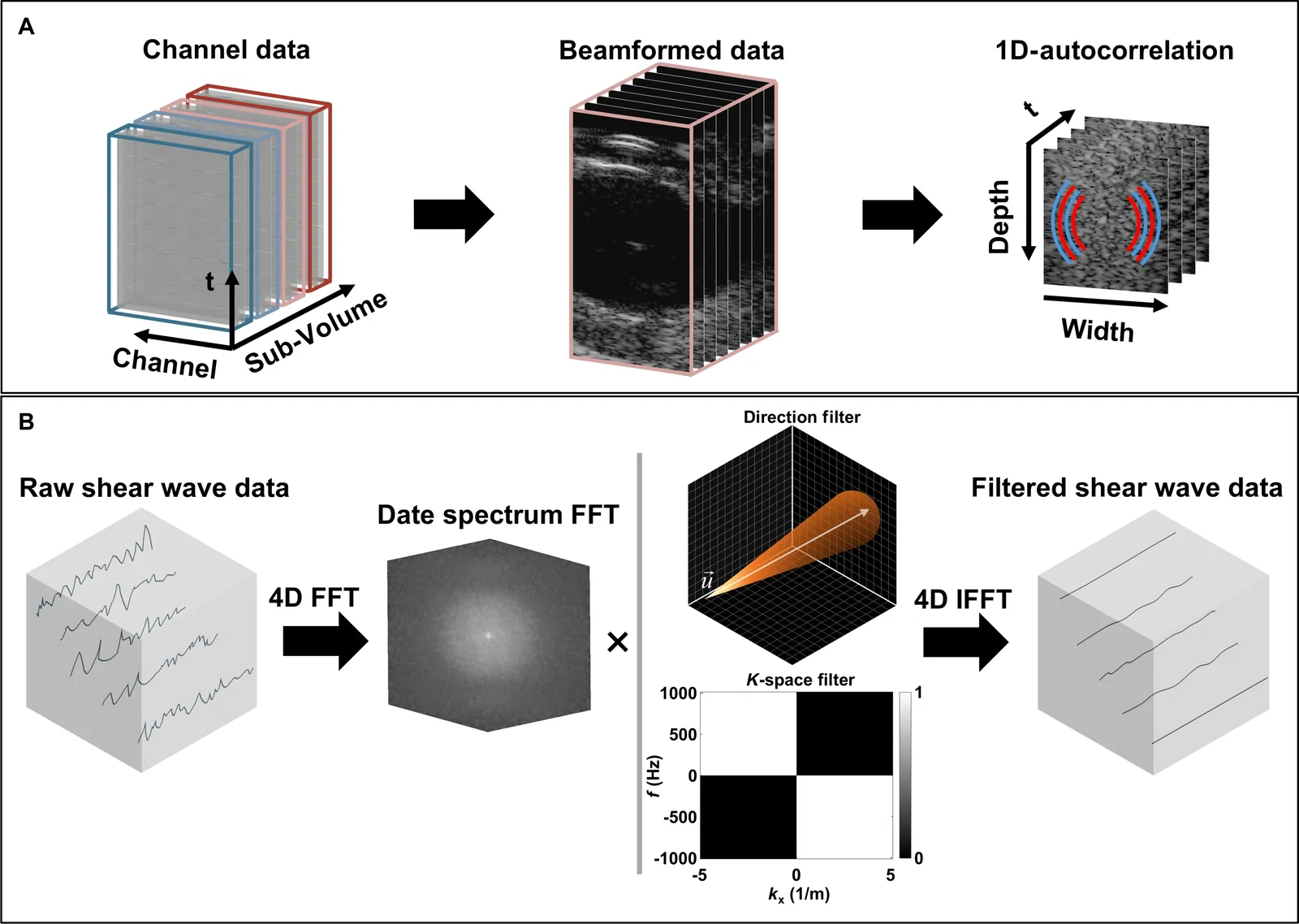
Data processing pipeline. (A) Subpanel data compounding and 1D autocorrelation processing. (B) 4D fast Fourier transform (FFT) and direction filtering.

**Fig. 4.**
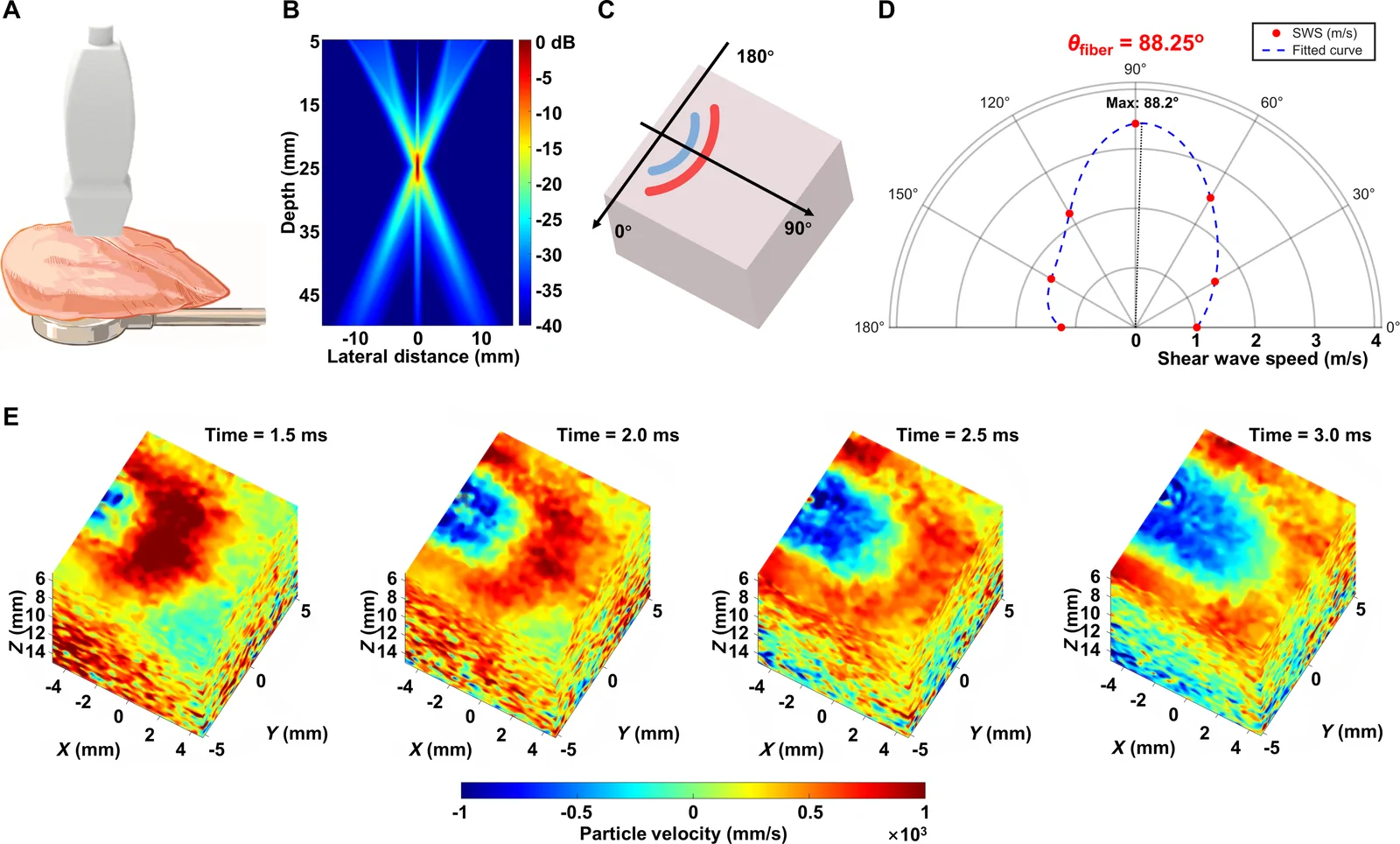
Ex vivo experimental results in chicken breast. (A) Schematic of the experimental setup. (B) Field II simulation results of the ring-shaped ultrasound transmitter [outer diameter (OD): 25 mm; inner diameter (ID): 10 mm; focal depth: 25 mm]. (C) Schematic of the coordinate system for fiber orientation estimation. (D) Muscle fiber orientation estimation results based on shear wave velocities measured in 7 directions. (E) Temporal evolution of shear wave propagation.

The data processing pipeline is shown in Fig. 3. The experiment setup is shown in Fig. 4A, where the 2D matrix array and the ring push transducer were placed symmetrically on opposite sides of the chicken breast. The parameters of the ring-shaped ultrasound transmitter are shown in Fig. S1. The ultrasound transmitter was applied to generate the ultrasound pushing beam to induce the shear propagation. The simulated acoustic field of the ring ultrasound transmitter is presented in Fig. 4B, and hydrophone test results are shown in Figs. S2 and S3. To enable the quantification of 3D shear wave velocity, a coordinate system was defined on the 4D shear wave dataset, as shown in Fig. 4C. The detailed values in each direction were shown in Table S1. Based on this coordinate system, the muscle fiber direction was estimated to be 88.25°. The propagation of the shear wave is shown in Fig. 4E.

### 3D shear wave velocity mapping of the rabbit cornea

**Fig. 5.**
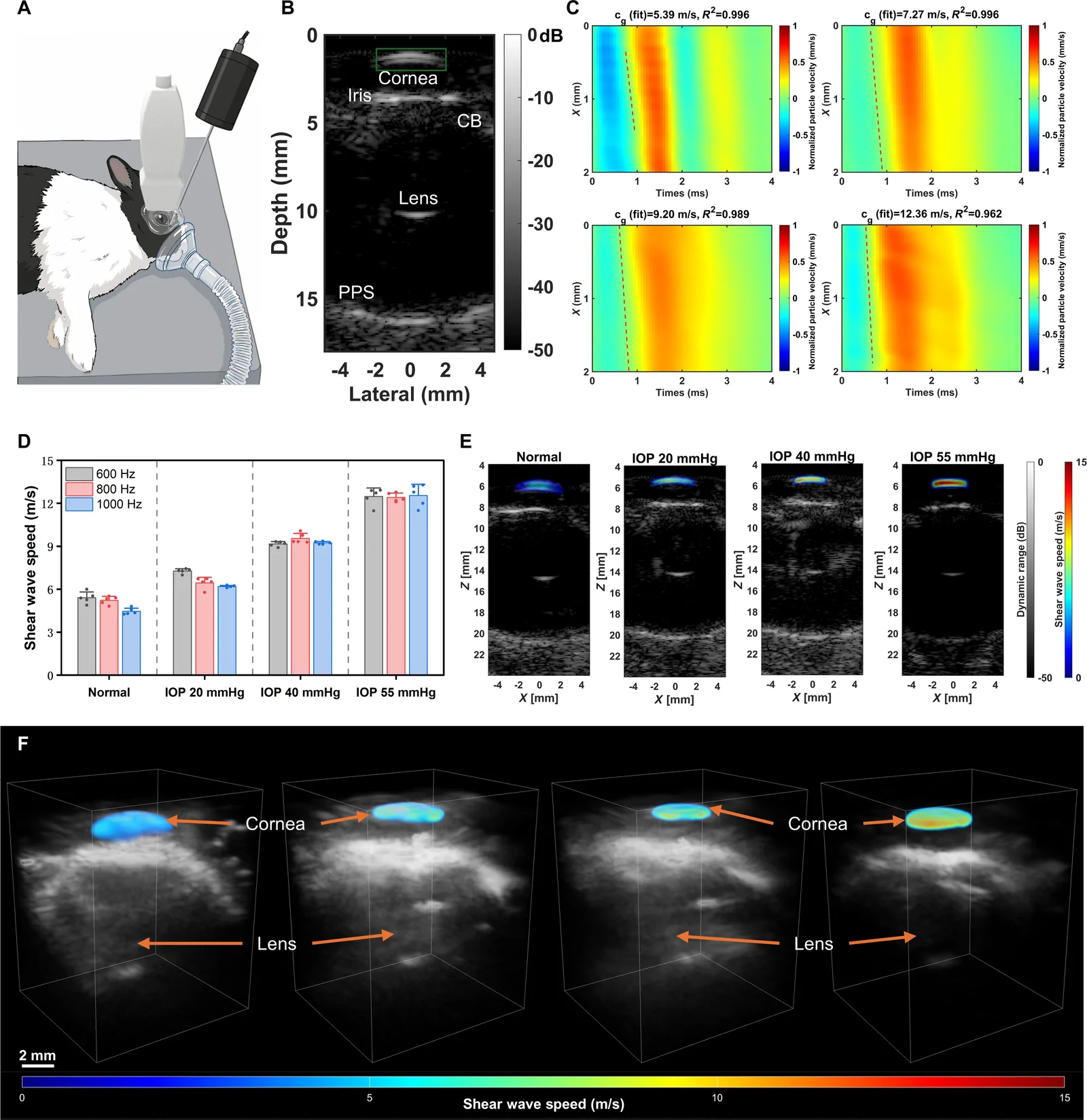
In vivo corneal elastography experiment in rabbit. (A) Schematic of the experiment setup. (B) B-mode image of the rabbit eye. (C) Spatial-temporal map under different intraocular pressure (IOP) at mechanical excitation frequency of 600 Hz. (D) Corneal shear wave velocity under different IOPs at mechanical excitation frequencies of 600, 800, and 1,000 Hz. (E) 2D shear wave velocity mapping. (F) 3D shear wave velocity mapping. CB, ciliary body; PPS, peripapillary sclera.

In vivo corneal elastography obtained from rabbit eyes under different IOP (intraocular pressure) conditions was measured. Figure 5A shows the experiment setup, and the corresponding B-mode image of the rabbit is presented in Fig. 5B. The spatial-temporal map in Fig. 5C demonstrated the differences at different IOP levels, where the slope of the wavefront represents the shear wave velocity. As IOP increased, the slope became steeper, indicating that the shear wave propagates faster and the corneal stiffness increases accordingly. The quantitative measurements under mechanical excitations of 600, 800, and 1,000 Hz are summarized in Fig. 5D. The measured shear wave velocity increased from 5.03 ± 0.51 m/s at normal state to 6.64 ± 0.56 m/s at 20 mmHg, 9.32 ± 0.21 m/s at 40 mmHg, and 12.45 ± 0.07 m/s at 55 mmHg. The corresponding trend in Fig. S4 demonstrates the correlation between shear wave velocity and IOP. The 2D shear wave velocity maps shown in Fig. 5E and the corresponding 3D volumetric maps in Fig. 5F provide direct visualization of the spatial distribution of corneal elasticity.

These results demonstrate that volumetric elastography can be achieved using a 2D matrix array without relying on a fully populated acquisition system with an equivalent channel count, which is often associated with higher cost. By using the proposed sequential acquisition strategy, we established the feasibility of this approach for volumetric SWE while maintaining practical system requirements. This method successfully differentiated tissue anisotropy and captured clinically relevant biomechanical changes in ocular tissues, specifically the increase in corneal stiffness associated with elevated IOP as in glaucoma-related conditions, without adding system costs compared with conventional 2D elastography.

## Discussion and Conclusion

In this study, we developed a sequential excitation-based framework for 4D SWE using a 2D matrix array, with the goal of reducing system requirements while maintaining high-quality volumetric elastography. Unlike fully connected matrix array methods that generally require higher channel counts and hardware complexity, the proposed strategy enables volumetric shear wave imaging on a practical acquisition system (e.g. Vantage 256) [16]. This design provides a cost-effective solution for 4D elastography and improves accessibility of volumetric imaging without relying on a fully connected element-to-channel system.

A key contribution of this work is the optimization of the transmit–receive sequence. Compared to our previous implementation, this sequence notably improves spatial resolution, particularly in the elevational direction. Notably, at a 10-mm depth, the PSF extent was reduced from 4.7*λ* to 2.1*λ*, corresponding to an improvement of 2.24-fold. This result highlights the importance of sequence design in matrix array-based volumetric imaging. Furthermore, this improvement in resolution is particularly important for SWE, as it allows for precise shear wave velocity calculations while maintaining high spatial resolution.

The proposed method was further validated in vitro, successfully achieving volumetric elastography and differentiating tissue anisotropy. These results indicate that the sequential excitation framework not only generates volumetric wave propagation maps but also captures mechanically relevant directional information from anisotropic tissues [25,33]. Building upon conventional 2D direction filters [3,34], we further implemented a 3D direction filter for volumetric shear wave analysis, which is important because of the 3D nature of shear wave propagation in anisotropic tissue that 2D analysis cannot fully characterize. By incorporating this direction filter into the volumetric domain, the proposed method provides a more comprehensive assessment of tissue mechanical properties.

The in vivo rabbit experiment further demonstrated the real-world applicability of this approach for ocular elastography [5]. In particular, 3D shear wave velocity mapping was demonstrated in the cornea, showing that this method can capture biomechanical information in biological tissue samples. Because corneal stiffness is closely associated with IOP and is highly relevant to ocular diseases such as glaucoma [35], these results suggest that volumetric SWE could provide a useful tool for monitoring IOP-dependent diseases in the eye, especially when traditional methods for measuring IOP cannot be performed. Compared with conventional 2D mapping [5], 3D shear wave velocity mapping offers a more complete representation of tissue structure and mechanical variation, which could be an advantage for characterizing complex tissue structures.

Despite these promising results, this study has several limitations. The primary trade-off of the sequential excitation strategy is reduced volume rate [16]. In the current implementation, the volume rate was approximately 0.5 Hz, corresponding to about 2 s for acquisition of a complete volume. This slow volume imaging speed may limit the application of the method in scenarios involving rapid motion. Nevertheless, the sequential approach provides a practical balance between hardware simplicity and volumetric imaging capability. Further work is needed to improve acquisition efficiency and reconstruction strategies. In addition, future studies are needed to validate the method in clinical trials to explore its translational potential in ophthalmic applications.

Taken together, these results demonstrate that sequential excitation with a 2D matrix array can serve as a practical and effective strategy for 4D SWE. The proposed framework not only improves the volumetric imaging quality through optimized sequence design but also enables anisotropy assessment and in vivo 3D shear wave velocity mapping without increasing system complexity. More importantly, it further demonstrates the first successful 3D shear wave velocity mapping of ocular tissue. This work therefore establishes the feasibility of cost-effective volumetric elastography and supports the future development for broader biomechanical and clinical applications.

## Materials and Methods

### Experimental and technical design

A programmable Vantage 256 system (Verasonics Inc., Kirkland, WA, USA) was used to transmit and acquire the ultrasound signal. A 32 × 32 elements matrix array (Matrix1024-8, Verasonics Inc., Kirkland, WA, USA) was divided into 4 subpanels (8 × 32 elements each) and connected to a 256-channel programmable ultrasound system via a 4-1 Multiplexing (UTA 1024-MUX adapter, Verasonics Inc.), as shown in Fig. 1B. A function generator (AFG3252C, Tektronix, Beaverton, OR, USA) was synchronized with the imaging system to provide external trigger signal and excitation control [30]. For the ARF-based SWE setup, the Vantage 256 system sent a trigger to the function generator. Then, a sinusoidal burst was produced and sent to the amplifier (75A250A, AMETEK Inc., PA, USA), which is used to drive a customized ring push transducer with a center frequency of 5.5 MHz (Fig. S1). The function generator was set to 5.5 MHz with 5,000 cycles for ARF excitation. For mechanical excitation SWE, the output of the function generator was connected to a power amplifier (Type 2718, Bruel & Kjaer, Duluth, GA, USA), and the amplifier signal was delivered to a shaker (#4810, Bruel & Kjaer, Duluth, Georgia, USA). The shaker rod was gently placed in contact with the ocular tissue near the corner of the eye to transmit mechanical vibrations. In this study, the mechanical excitation frequencies were set to 600, 800, and 1,000 Hz.

### Acquisition sequence

The 2D matrix array is composed of 1,024 elements partitioned into 4 subpanels [31], each containing 256 elements arranged in an 8 × 32 layout. The element pitch was 0.3 mm in both lateral and elevational directions. Due to the 0.6-mm gap between the adjacent subpanels (Fig. 1B), the field of view was 9.3 mm × 10.2 mm. These gaps introduce discontinuities in the effective aperture, which can degrade the elevational image quality. To address this effect, the cross-panel acquisition scheme was employed as shown in Fig. 2. With 4-1 multiplexing, a maximum of 256 elements could be controlled in a single transmit–receive event. The multiplexing switch was connected to elements in the sequence i, i+256, i+512, and i+768. Although a variety of multiplexing strategies have been proposed to improve elevational resolution [36], elastography must account for the constraints of volumetric frame rate and potential shear wave interference arising from sequential excitations. To mitigate the impact of multiplexing-induced subpanel switching delays, we used the sequential excitation scheme, in which an independent shaker or ARF excitation was applied for each subpanel acquisition. Following each excitation, ultrasound data were received using the corresponding subpanel according to the sequence as shown in Fig. 2A. After completion of the acquisition, the system switched to the next subpanel, and the excitation and acquisition process was repeated. A 250-ms interval was maintained between successive excitations to allow the tissue to return to its resting state before the next measurement. Each beamformed 3D volume was reconstructed from 8 subpanel acquisitions. For each subpanel acquisition, radiofrequency (RF) data were collected using 5-angle plane-wave imaging. The acquired RF channel data from all transmit angles and subpanel acquisitions were then beamformed using coherent plane-wave compounding beamforming [14]. Parmeter settings are listed in Table S2.

### Data processing

The data processing pipeline is shown in Fig. 3. First, the acquired channel data were reconstructed using a beamforming delay-and-sum method. As illustrated in Fig. 3A, the beamformed data were organized into a 4D array $D\left(z,x,y,t\right)$, where z,x,y, and t represent depth, lateral, elevational, and temporal dimensions, respectively. The shear wave propagation was then estimated from the beamformed data using a 1D autocorrelation method [37].

A volumetric directional filter was then applied in the 4D frequency domain [38,39], as illustrated in Fig. 3B. First, the shear wave data $D\left(z,x,y,t\right)$ were zero-padded and transformed into $\left(k_{z},k_{x},k_{y},f\right)$ domain using a 4D Fourier transform:

$$\begin{array}{c} D\left(k_{z},k_{x},k_{y},f\right)=F_{4D}\left\{D\left(z,x,y,t\right)\right\} \end{array}$$ (1)

where $k_{z}$, $k_{x},$ and $k_{y}$ are the spatial frequencies along the depth, lateral, and elevational directions, respectively. f is the temporal frequency.

The directional filter was designed to preserve shear wave components propagating along the desired direction. The target propagation direction was defined by an azimuth angle ϕ and an elevation angle θ[24]. The corresponding unit direction vector was defined as:

$$u=\left[\begin{array}{c} u_{z}\\ u_{x}\\ u_{y} \end{array}\right]=\left[\begin{array}{c} \text{sin}\;\theta \\ \text{cos}\;\theta \;\text{cos}\;\phi \\ \text{cos}\;\theta \;\text{sin}\;\phi \end{array}\right]$$ (2)

here, the azimuth angle ϕ specifies the vector in x−y plane, where ϕ=0° and ϕ=90° correspond to the +x and +y directions. θ represents the elevation angle such that θ=0° corresponds to the direction entirely within the x−y plane and θ=90° corresponds to the +z direction.

The directional filtering process consisted of 2 components, a spatial filter and a spatiotemporal filter.

The spatial filter was then defined as:

$$H_{1}\left(k_{z},k_{x},k_{y}\right)=\left\{\begin{array}{c} {\left(\frac{u\cdot k}{\left\|k\right\|}\right)}^{q},\;u\cdot k\geq 0\\ 0,\;u\cdot k\geq 0 \end{array}\right.$$ (3)

where *k* is the spatial frequency vector $k={\left[k_{z},k_{x},k_{y}\right]}^{T}$. q controls the angular selectivity of the filter. A larger q yields a narrower angular passband.

The spatiotemporal filter was defined as follows:

$$H_{2}\left(k_{z},k_{x},k_{y},f\right)=\left\{\begin{array}{c} 1,\;\left(u\cdot k\right)f\leq 0\\ 0,\;\left(u\cdot k\right)f>0 \end{array}\right.$$ (4)

The final direction filter was obtained by combining $H_{1}$ and $H_{2}$:

$$H\left(k_{z},k_{x},k_{y},f\right)=H_{1}\left(k_{z},k_{x},k_{y}\right)H_{2}\left(k_{z},k_{x},k_{y},f\right)$$ (5)

Finally, the filtered shear wave data were obtained in the frequency domain by applying the final direction filter, followed by inverse 4D Fourier transform:

$$\begin{array}{c} D_{\text{filt}}\left(z,x,y,t\right)=F^{-1}\left\{D\left(k_{z},k_{x},k_{y},f\right)H\left(k_{z},k_{x},k_{y},f\right)\right\} \end{array}$$ (6)

### Shear wave speed calculation

The filtered shear wave propagation data were represented as a 4D matrix $D\left(z,x,y,t\right)$, where z is the axial direction, x is the lateral direction, y is the elevational direction, and y is the time. Because the shear wave is filtered in the lateral (x) direction, the 4D matrix data are processed as a 3D data block on each y plane:

$$D_{y}\left(z,x,t\right)=D\left(z,x,y,t\right)$$ (7)

where $y\in \left\{1,\ldots ,N_{y}\right\}$, and the shear wave speed was calculated on each y plane.

For each y plane, the temporal signal was cut from 0 to 5 ms after excitation, which retained the main propagation period and reduced the influence of noise signal. The cropped signal was then interpolated along the temporal dimension using spline interpolation. The interpolation factor was set to F=8, which yields the up-sampled shear wave propagation data $\tilde{D}\left(z,x,\tilde{t}\right)$ with sampling step $\tilde{t}=t/F$.

At each pixel $\left(z_{m},x_{n}\right)$ in the selected y plane, a local 2D square window $\Omega \left(z_{m},x_{n}\right)$ was used to estimate the shear wave speed along x. The width of $\Omega \left(z_{m},x_{n}\right)$ is 2h+1, where $h=\left[\left(L-W_{s}\right)/2\right]$. For every position $\left(z_{i},x_{j}\right)\in \Omega$, 2 temporal signals were extracted from $\tilde{V}$:

$$\begin{array}{l} S_{1}\left(\tilde{t}\right)=\tilde{D}\left(z_{i},x_{j}-W_{s}/2,\tilde{t}\right)\\ S_{2}\left(\tilde{t}\right)=\tilde{D}\left(z_{i},x_{j}+W_{s}/2,\tilde{t}\right) \end{array}$$ (8)

where L defines the total lateral processing aperture in pixels, whereas $W_{s}$ defines the lateral spacing in pixels between the 2 signal locations used for shear wave propagation time-of-flight estimation. The time lag $\tau \left(z_{i},x_{j}\right)$ between $S_{1}$ and $S_{2}$ was estimated by using the normalized cross-correlation. The local shear wave velocity is then calculated:

$$v\left(z_{i},x_{j}\right)=\frac{W_{s}\cdot d_{x}}{\tau \left(z_{i},x_{j}\right)}$$ (9)

where $d_{x}$ is the lateral pixel spacing, which converts pixel to distance.

The final shear wave speed at the center pixel $V\left(z_{m},x_{n}\right)$ was estimated by the square of cross-correlation coefficients $c\left(z_{i},x_{j}\right)$ and the distance $r\left(z_{i},x_{j}\right)$ to the center pixel [3]:

$$\begin{array}{c} \begin{array}{c} V\left(z_{m},x_{n}\right)=\sum_{i=m-h}^{m+h}\sum_{j=n-h}^{n+h}\left\{v\left(z_{i},x_{j}\right)\cdot \frac{c{\left(z_{i},x_{j}\right)}^{2}/r\left(z_{i},x_{j}\right)}{\sum_{i=m-h}^{m+h}\sum_{j=n-h}^{n+h}c{\left(z_{i},x_{j}\right)}^{2}/r\left(z_{i},x_{j}\right)}\right\} \end{array} \end{array}$$ (10)

The 3D shear wave speed volume $U\left(z,x,y\right)$ was calculated using a 3D distance-weighted averaging method. For each voxel, a cubic kernel centered at $\left(z,x,y\right)$ was selected from the origin shear wave speed volume $V\left(z,x,y\right)$. The shear wave speed at the center voxel was estimated by calculating the weighted average of all valid voxels within the cube:

$$U\left(z,x,y\right)=\frac{\sum_{i,j,k\in \Omega }w\left(i,j,k\right)V\left(z+i,x+j,y+k\right)}{\sum_{i,,j,k\in \Omega }w\left(i,j,k\right)}$$ (11)

where $V\left(z+I,x+j,y+k\right)$ represents the original shear wave speed at the neighboring voxel within the cubic region Ω, and $w\left(i,j,k\right)$ is the distance weighting factor. $w\left(i,j,k\right)=1/\sqrt{i^{2}+j^{2}+k^{2}}$.

### Phantom study and ultrasound imaging

For characterization of the ultrasound imaging performance, the phantom experiments were performed in B-mode imaging mode. A 20-μm tungsten wire phantom was used for spatial resolution assessment. The wires were placed at depths of 10, 14, 18, and 22 mm. Imaging was performed using the 2D matrix array in both the azimuthal and elevational directions to calculate the axial, lateral, and elevational resolutions. To further evaluate the PSF, a 50-μm needle target was imaged in the x−y plane. For each reconstructed target, the image intensity was normalized to the peak intensity, and the axial, lateral, and elevational intensity profiles through the target center were extracted for spatial resolution analysis. The FWHM was calculated and used as the measure of spatial resolution. Representative results are shown in Fig. 2C and D.

### Ex vivo chicken breast model

Due to multiplexing, ARF excitation cannot be applied to the 2D matrix array. Therefore, ARF-based SWE was performed using a customized ring push transducer. As shown in Fig. 4A, the 5.5-MHz ring push transducer and 2D matrix array were placed on opposite sides of the chicken breast sample. Before the start of each subvolume acquisition, the Vantage 256 system sent a trigger out signal to the function generator to drive the push transducer and generate a focused push. A time interval of 250 ms was applied to allow the tissue to recover to its resting state. Since 8 sequential acquisitions were required to reconstruct one complete volume, the total acquisition time for each volume was approximately 2 s. To characterize the directional dependence of shear wave propagation, a coordinate was defined on the volumetric dataset acquired. Within the selected analysis plane, the angular range from 0° to 180° was sampled at 30° intervals, as shown in Fig. 4C. The shear wave velocity was then calculated along each sampled direction.

### Animals and study approval

For the in vivo ocular elastography experiments, shear waves were generated using an external mechanical shaker rather than ARF excitation to minimize the risk of tissue damage. Dutch belted rabbits (4 month) were anesthetized with intramuscular ketamine (35 mg/kg) and xylazine (5 mg/kg). During anesthesia, heart rate, respiratory rate, oxygen saturation, body temperature, and noninvasive blood pressure were recorded every 5 min. Additional anesthetic was administered as needed according to physiological status. An infusion line and a pressure sensor were inserted to maintain and monitor the IOP [5]. The rabbit study was approved by the University of Southern California Institutional Animal Care and Use Committee (IACUC) under protocol no. 21345.

## Data Availability

The data of animal study that supports the findings of this study are available upon reasonable request from the corresponding author.
