# Supplementary material for: Four-Dimensional Sequential Elastography for Ocular Tissue Using 2D Matrix Array
Source: BME Front. 2026 Sep 9;7:0307. doi: 10.34133/bmef.0307 (PMC13554413; doi:10.34133/bmef.0307)
Supplement: Supplementary file 1 [file bmef.0307.f1.docx]

**Supplementary Figures**

**Figure S1**. Ring push transducer parameters. (a) The picture of transducer. (b) Frequency spectrum. (c) Impendence and phase results. (d) Pulse-echo results.

**Figure S2**. Acoustic field in the XZ plane of the pushing transducer measured by hydrophone.

**Figure S3**. Transmitting voltage response (TVR) of the pushing transducer.

**Figure S4**. Shear wave speed at different IOP levels under excitation frequencies of 600, 800, 1000 Hz. (a) 600 Hz. (b) 800 Hz. (c) 1000 Hz.

**Supplementary Tables**

**Table S1.** Direction angles with shear wave velocity

| Direction Angle (Degree) | Shear Wave Velocity (m/s) |
| --- | --- |
| 0 | 1.03 ± 0.02 |
| 30 | 1.53 ± 0.07 |
| 60 | 2.51 ± 0.09 |
| 90 | 3.42 ± 0.32 |
| 120 | 2.21 ± 0.15 |
| 150 | 1.63 ± 0.12 |
| 180 | 1.24 ± 0.03 |

**Table S2.** Parmeter settings

|  | 2D Matrix Array 1024-8 MHz |
| --- | --- |
| Transmit center frequency (MHz) | 7.8125 |
| Number of elements | 1024 |
| Pitch (mm) | 0.3 |
| Transmission type | Plane wave |
| Steering Angles | (0°, 0°), (-5°, 0°), (5°, 0°), (0°, -5°), and (0°,5°) |
| Pulse Repetition Frequency (Hz) | 10k |
| Volume Rate (Hz) | 0.5 |
| Total number of frames | 300 |
